# Real-World Survival in Patients with Metastatic Melanoma after Discontinuation of Anti-PD-1 Immunotherapy for Objective Response or Adverse Effects: A Retrospective Study

**DOI:** 10.1155/2021/5524685

**Published:** 2021-04-27

**Authors:** Julie Valentin, Thomas Ferté, Valérie Dorizy-Vuong, Léa Dousset, Sorilla Prey, Caroline Dutriaux, Anne Pham-Ledard, Marie Beylot-Barry, Emilie Gérard

**Affiliations:** ^1^Department of Dermatology, Hôspital Saint André, University Hospital of Bordeaux, Bordeaux, France; ^2^Bordeaux Hospital University Center, Pôle de Santé Publique, Service D'information Médicale, Unité Informatique et Archivistique Médicales, F-33000 Bordeaux, France; ^3^Université de Bordeaux, INSERM U1035, F-33076 Bordeaux, France; ^4^INSERM U1053, Bordeaux Research in Translational Oncology, Team 3 Oncogenesis of Cutaneous Lymphomas, Université de Bordeaux, Bordeaux, France

## Abstract

**Objective:**

Anti-PD-1 has dramatically improved the survival of patients with advanced melanoma. However, there is a lack of data on maintenance of the response after treatment discontinuation. We aimed to evaluate the progression-free survival (PFS) of patients with metastatic melanoma after anti-PD-1 interruption for objective response (OR) or limiting toxicity during clinical trials.

**Methods:**

All patients with advanced melanoma who stopped single-agent anti-PD-1 antibodies for objective response or toxicity were included between April 2014 and January 2019 in our institution (data cut-off, September 10th, 2019). Clinical and biological factors associated with relapse were studied.

**Results:**

The median follow-up after introduction of treatment was 36.5 months [4.6–62.4], and the median follow-up after discontinuation of treatment was 15.7 months (2.5–45.1). Out of 65 patients, 28 patients stopped immunotherapy for limiting adverse effects (AEs) (43.1%), 25 for complete response (CR) (38.4%), and 12 for partial response (PR) or long-term stable disease (SD) (18.5%). Twelve patients relapsed (18.5%) after a median time of 9 months [1.9–40.9 months]. Seven relapsed after discontinuation for AEs, 3 after discontinuation for CR, and 2 after discontinuation for PR/SD. The median PFS after therapy discontinuation was not reached. No statistical association was found between recurrence and age, sex, increased LDH, BRAF status, presence of brain metastases, previous treatments, radiotherapy, or time on anti-PD-1 treatment.

**Conclusion:**

This cohort shows a global recurrence rate of 18.5% and confirms a long-lasting response after anti-PD-1 cessation regardless of the cause of discontinuation.

## 1. Introduction

The management of patients with metastatic melanoma has been revolutionized during the last decade by the emergence of new therapies, such as BRAF and MEK inhibitors and immune check-point inhibitors [1, 2]. Melanoma is considered to be one of the most immunogenic solid tumors [3, 4]. Strategies to stimulate the antitumor immune response are critical, especially in patients without BRAF mutations. The programmed cell death-1 (PD-1) receptor is expressed on activated T cells, B cells, macrophages, regulatory T cells, and natural killer cells. The anti-PD-1 monoclonal antibodies, pembrolizumab and nivolumab, block binding of PD-1 to its ligands PD-L1 and PD-L2 [5].

There is no recommendation on the optimal duration of immunotherapy by PD-1 inhibitors. These missing data are crucial in daily practice, as patients often request to cease therapy after objective response. Other concerns emerge, such as the immune-related toxicities management and the benefit-risk ratio of a prolonged treatment or the financial burden [6]. In most clinical trials, treatment was discontinued according to arbitrary durations. In the KEYNOTE-001 trial, pembrolizumab duration was set for 2 years or discontinuation after complete response (CR) if patients received treatment for at least 6 months and had received at least 2 treatment infusions after the assessment of CR [7].

In addition, 3-year, 4-year, and 5-year survival data from these initial cohorts of patients who discontinued treatment show encouraging results of long-lasting efficacy [8–10]. In KEYNOTE-001, the 24-month progression-free survival rate was 89.9% in the patients who discontinued treatment for CR. In KEYNOTE-006 (post hoc 5-year data), regarding the patients who discontinued after 2 years of pembrolizumab, 24-month progression-free survival (PFS) was 78.4%. 24-month overall survival (OS) was 95.9%, and 36-month OS was 93.8%. Moreover, in the patients with CR who discontinued pembrolizumab early, 24-month PFS was 86.4%. In the CheckMate-067 trial, 58% of the patients who initially received nivolumab alone and who were not under treatment were still alive at 5 years.

In the present real-life study, we aimed to assess the PFS in patients with metastatic melanoma after discontinuation of anti-PD-1 antibodies for objective response (OR) (CR or partial response (PR)), durable stable disease (SD), or for limiting adverse events (AEs). In addition, we analysed potential predictive factors associated with relapses.

## 2. Materials and Methods

### 2.1. Study Design and Patients

We conducted an observational, retrospective, monocentric study (University Hospital of Bordeaux, France). Data were collected from the medical files and then were anonymized and protected for the analysis during the study.

**Figure 1:**
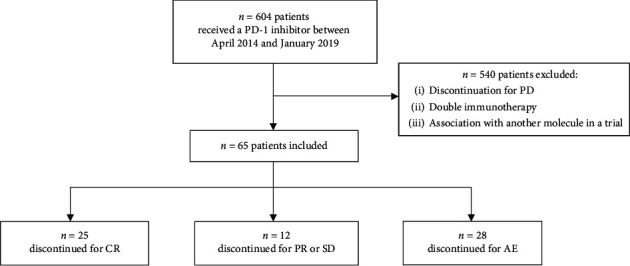
Flow chart of patients selection. Abbreviation: PD, disease progression; CR, complete response; PR, partial response; SD, stable disease; AE, adverse event.

We selected all consecutive patients with metastatic or unresectable melanoma treated with anti-PD-1 monotherapy (whatever the line) from April 2014 to January 2019. Patients were included if they had discontinued immunotherapy for OR, SD, or AEs and if they did not receive another subsequent systemic treatment for their metastatic melanoma. Patients who discontinued treatment for progression and those who received combination of anti-PD-1 with another treatment (ipilimumab or another molecule in a clinical trial) were excluded (Figure 1). All patients provided written informed consent to participate in this study. This study was approved by the ethics committee of Bordeaux University (GP-CE2020-11).

### 2.2. Clinical Analyses

Clinical and biological baseline parameters were assessed at the time of therapy introduction (Table 1).

Patients received either pembrolizumab 2 mg/kg every 3 weeks or nivolumab 3 mg/kg every 2 weeks or 480 mg every 4 weeks. CT scans were performed every 12 weeks as a routine procedure, and responses were evaluated by the Response Evaluation Criteria in Solid Tumors, version 1.1 (RECIST 1.1).

At the time of treatment discontinuation, patients were classified into three subgroups according to the cause of treatment discontinuation: CR, PR/SD, or AEs if treatment was discontinued for limiting toxicities. If the patients discontinued treatment because of limiting toxicities before the first CT scan during treatment, they were considered as SD. Responses were assessed at the time of discontinuation and during follow-up. During follow-up, relapse or disease progression (PD) were recorded using RECIST 1.1.

PFS was defined as the time between immunotherapy discontinuation and cancer recurrence/progression or last follow-up in patients who did not experience a relapse/progression. Remaining patients were right censored on September 10, 2019. The follow-up after introduction of treatment was the time between the first infusion of treatment and the last follow-up. The duration of follow-up was the time between the discontinuation of anti-PD-1 antibodies and the last follow-up. Time on treatment (TOT) was the interval between the introduction of immunotherapy and the last administration of treatment. Time from therapy initiation to the best response was defined as the interval between the treatment introduction and the time of the best response (CR, PR, or SD). Common Terminology Criteria for Adverse Events Criteria, Version 4.03, were used to rate immune-related AEs (irAEs).

### 2.3. Statistical Analysis

The characteristics of patients were described with median and interquartile range for quantitative variables and numbers and percentages for qualitative variables.

Progression-free survival, defined as the time between immunotherapy discontinuation and cancer recurrence/progression or last follow-up, was estimated using the Kaplan–Meier method.

A multivariate Cox model was built to look for factors associated with PFS. Treatment duration and variables reaching the 0.25 level of significance in univariate analysis were included in the multivariate analysis. Proportional hazards assumption was checked by examining the Schoenfeld residuals. Log linearity assumption was checked using a fractional polynomial method. Kruskal–Wallis and Fisher tests were used for subgroup comparison. Statistical analyses were realised using the R v3.4.4 software and the package survival.

## 3. Results

### 3.1. Patient Characteristics and Treatment

Six hundred and four patients with metastatic melanoma receiving single-agent anti-PD-1 immunotherapy were identified. Out of these, 65 (10.7%) stopped for another reason than progression and were included. Median and range of follow-up after introduction of treatment was 36.5 months [4.6–62.4]. Baseline characteristics are presented in Table 1.

Among 65 patients who stopped anti-PD-1 immunotherapy, 25 patients (38.4%) discontinued treatment for CR, 12 patients (18.5%) for PR or SD, and 28 patients (43.1%) because of AEs. Overall median and range of TOT was 14.1 months [0.7–51.2] and 16.8 months [7.6–34.9] in the CR subgroup and 21.2 months [10.1–51.2] in the PR/SD patient subgroup. Median TOT was shorter in the AE subgroup (7.2 months [0.7–30.2]; *p* < 0.001). Median and range time to best response was 6.2 months [2.5–21.4] in the CR subgroup and 4.9 months [2.8–27.8] in the PR/SD subgroup.

### 3.2. Outcomes

**Figure 2:**
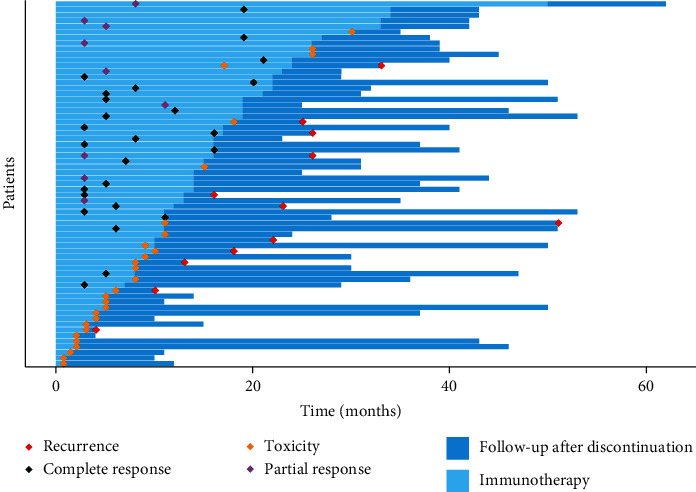
Follow-up of patients who discontinued anti-PD-1, University Hospital of Bordeaux, 2019. Of the 65 patients included, 38% (*n* = 25) discontinued treatment for CR, 18% (*n* = 12) for PR or SD, and 43% (*n* = 28) because of AEs.

**Figure 3:**
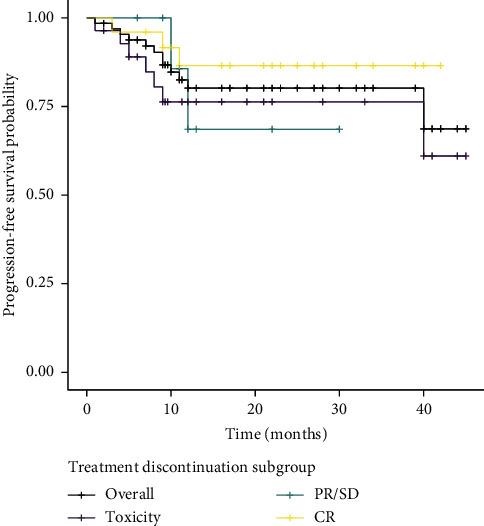
Kaplan–Meier curves of progression-free survival (PFS) from the discontinuation for other reasons than PD (*n* = 65) and according to the confirmed response during treatment, University Hospital of Bordeaux, 2019. The overall subgroup represents all the patients included in the cohort (*n* = 65). The subgroup CR represents the 25 patients who discontinued for CR, while 12 patients were in the PR/SD subgroup and 28 were in the toxicity subgroup. The hash marks designate patients who were censored at that time point. There was no statistically significant association between the cause of treatment discontinuation and PFS (*p* > 0.5).

At the end of the study, 12 patients (18.5%) had experienced a relapse after anti-PD-1 antibody discontinuation after a median time and range before a relapse of 9 months [1.9–40.9]. After a median follow-up after discontinuation of 15.7 months [2.5–45.1], 81.5% of the patients did not experience a relapse (Figure 2). 25% patients who discontinued for AEs relapsed after 7.1 months [1.9–40.9], while 16.7% from the PR/SD subgroup and 12% from the CR subgroup relapsed after 11.9 months [10.9–12.9] and 9.3 months [4–11.8], respectively (Figure 3).

At the end of the study, 62 patients were still alive. One patient who did not experience a relapse died from another cause than melanoma. Two patients who had relapsed died: 1 related to progression disease and 1 for other reason than progression disease or AE.

**Figure 4:**
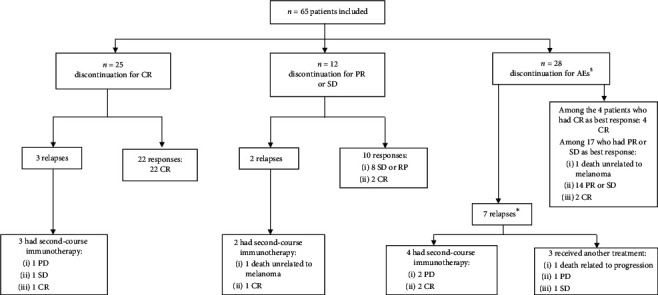
Outcomes of the 65 patients included according to subgroup classification based on the cause of treatment discontinuation and outcomes after second-course anti-PD-1. Of the 65 patients who discontinued anti-PD-1 for other reasons than PD, 12 patients relapsed and 9 of them received a second-course anti-PD-1. $ comprises 4 CR and 24 PR or SD as best response while on treatment. *∗*All patients had PR or SD as best response.

Outcomes according the patients' subgroup according to the cause of treatment discontinuation are presented in Figure 4. Regarding the 37 patients who stopped treatment for controlled disease (25 CR and 12 PR/SD), 5 patients experienced a relapse. Of those, 3 patients were in CR and 2 in PD/SD. Median time and range before relapse was 9.3 months [4–11.8] in the CR group, and 11.9 months [10.9–12.8] in the PR/SD group. At the last follow-up, 26/37 patients were in CR (including 3 patients in PR/SD who reached CR during follow-up), 9 patients were in PR/SD, 1 patient experienced a PD, and 1 patient who relapsed had died.

Limiting treatment-related AEs that led to discontinuation occurred in 28 patients within a median time of 7.2 months [0.7–30.2] after the first infusion of anti-PD-1 antibodies. At the time of discontinuation, 24/28 patients were in PR/SD and 4 in CR. Seven patients experienced a relapse after a median time and range of 7.1 months [1.9–40.9]. All of them were in PR/SD after discontinuation. At the last follow-up, 23/28 patients still had a controlled disease (8 CR and 15 PR/SD), 3 patients experienced PD and 2 had died, and 1 related to PD. 4 patients reached CR during follow-up after discontinuation (1 SD and 3 PR).

Of the 12 patients who relapsed, 5 were in their first line of treatment, 3 patients had received prior ipilimumab, 2 had received prior BRAF and MEK inhibitors, and 2 had received both prior ipilimumab and prior targeted therapies. Six (50%) patients had relapsed in known metastatic sites, and 6 patients had new lesions. Of those, 2 were in the CR group, 1 in the PR/SD group, and 3 in the AE group. Characteristics of the patients who relapsed are presented in Table 2.

### 3.3. Immune-Related Adverse Events

Observed limiting toxicities that led to discontinuation included hepatic cytolysis (*n* = 6; 21.4%), colitis (*n* = 4; 14.3%), pneumonitis (*n* = 4; 14.3%), nephritis (*n* = 2; 7.1%), hypophysitis (*n* = 2; 7.1%), uveitis (*n* = 2; 7.1%), rheumatoid arthritis (*n* = 1; 3.6%), celiac disease (*n* = 1; 3.6%), gastritis (*n* = 1; 3.6%), polyradiculoneuritis (*n* = 1; 3.6%), asthenia (*n* = 1; 3.6%), thyroiditis (*n* = 1; 3.6%), hemophagocytosis (*n* = 1; 3.6%), and erythroblastopenia (*n* = 1; 3.6%). Grade 3 or grade 4 toxicities occurred in 18 patients who discontinued for AEs (64.3%).

### 3.4. Second-Course Immunotherapy

Nine out of 12 patients who relapsed received a second course of immunotherapy (response to the first course of treatment before discontinuation was CR for 3 of them and PR/SD for 2 of them and 4 discontinued for AEs). Out of the 3 patients who discontinued first-course treatment for CR, 2 are still treated with second-course immunotherapy (1 CR and 1 SD), and 1 experienced PD after retreatment. For the 2 patients who stopped for PR/SD, 1 is still on second-course treatment and 1 died of infectious diverticulitis. Of the 4 patients who had a second course of immunotherapy after discontinuation for AEs, one is now in CR after metastectomy without any therapy, while the 3 others are still receiving treatment (1 CR and 2 PD who will receive complementary radiotherapy).

### 3.5. Risk of Relapse

The potential prognostic baseline factors explored per multivariate analysis did not show a statistically significant association with relapse (Table 3). Moreover, time on treatment and type of response to the first treatment were not statistically associated with the risk of relapse (Table 4). PFS was not influenced by the cause of treatment discontinuation (Figure 3).

## 4. Discussion

In our study, after a median follow-up after treatment discontinuation of 15.7 months, 81.5% of patients did not experience any disease progression. Our data from daily practice are similar to that of previous publications, but our largest subgroup constated of patients who discontinued for AEs (43%). Schvartsman reports a median disease-free survival of 16 months in 89% of 75 patients who discontinued treatment for maximal clinical benefit or toxicities [11]. In the largest real-life cohort describing outcomes after anti-PD-1 cessation, 22% of PD after 18 months of follow-up (*n* = 185) was reported by Jansen et al. [12] and 18% of PD after 20 months of follow-up in the KEYNOTE-006 trial [13].

Even though limited data are available, the risk of recurrence is supposed to be low in patients who have stopped immunotherapy for CR [10]. In our cohort, only 3 patients in CR (12%) relapsed after anti-PD-1 cessation. Median TOT in this subgroup was 16.8 months. Even if we could not show a statistical link, it seems there is a tendency for patients who discontinued for CR to experience less relapse (Figure 3). In KEYNOTE-001, the 5-year follow-up of the patients who discontinued for CR showed a 60-month PFS of 63% [14]. Durable response to anti-PD-1 immunotherapy in patients in CR has also been reported in real-life studies. Ladwa and Atkinson reported 3 relapses amongst 29 patients who achieved CR after a median follow-up after discontinuation of 8 months [15]. Nguyen et al. reported 10.4% (*n* = 19) relapse amongst a group of 75 patients after a median time after anti-PD-1 discontinuation of 14 months [16]. Jansen et al. reported a higher risk of progression among the CR group treated with anti-PD-1 for less than 6 months, but there was no association between relapsing and a TOT superior to 6 months [12]. These data suggested that at least one year on treatment in the case of CR is required to experience a prolonged response.

In contrast, cessation of anti-PD-1 immunotherapy in patients in PR or SD questions: what is the optimal duration of treatment? In our cohort, the risk of relapse was not significantly different between the CR group and the PR/SD group (*p*=0.5). It may be explained by the limited size of the study. Of the 12 patients who discontinued treatment for PR or SD, 2 experienced a relapse (16.6%). Jansen et al. reported that prognosis in PR patients is different from that in CR patients with a higher risk of PD (34%), but the TOT was shorter than that found in our report (15 months for PR and 14 months for SD) [12]. In our cohort, patients who stopped immunotherapy for PR or SD had the longest treatment exposure (21.2 months). SD and PR cases may need to be treated for as long as possible. Tan et al. suggest that FDG-PET imaging after 1 year of anti-PD-1 antibodies could help predict long-term outcomes compared to CT imaging, especially for patients who experienced PR or SD [17, 18].

In our real-life study, AEs were the first reason for anti-PD-1 discontinuation (43.1%). Comparing our findings with previous reports with similar populations (discontinuation for other reason than PD, including toxicities), the incidence of discontinuation because of AEs is similar to Schvartsman's study (*n* = 34/75; 45.3%) [11] but higher than described by Gauci (*n* = 9/39; 23%) [19]. In our cohort, this group of patients seemed to have the worst PFS in the first nine months following discontinuation, but PFS was then stable till the 40^th^ month of follow-up (Figure 3), suggesting they had a clinical remnant benefit to anti-PD-1 immunotherapy. After cessation, 25% relapsed, and 2 patients died (1 because of PD). At the last follow-up, 23/28 patients still had a controlled disease.

In the KEYNOTE-001 study, seven patients had relapsed after discontinuing treatment and 4 of them received a second course of pembrolizumab with 50% OR (*n* = 2) (14). Nomura and colleagues reviewed 8 patients retreated with nivolumab, with 62% OR (*n* = 5) [20]. Betof Warner reported that, in the largest cohort to date, 34 patients received a second-course anti-PD-1 monotherapy, but only 5 patients (15%) responded [21]. In our cohort, 9 out of the 12 patients who relapsed received a second course of anti-PD-1 antibodies, of which 4 (44%) were patients who discontinued for AEs, without any toxicities. After retreatment, 5 of them had a controlled disease (56%).

The median time to relapse was 9 months after anti-PD-1 cessation. Close follow-up is required during the first year after anti-PD-1 cessation. In our study, the statistical power to identify factors associated with relapse after discontinuation is low. Moreover, on univariate analysis, the risk of relapse did not increase significantly with shorter TOT (*p*=0.178), even though TOT was statistically different between the 3 subgroups (*p* < 0.001). Discontinuation after PR/SD response to a first anti-PD-1 course compared to discontinuation for AEs was not associated with the risk of relapse (*p*=0.445). These data suggest that the immune response may also depend on the individually acquired resistance mechanisms to immunotherapy. Different resistance mechanisms to anti-PD-1 antibodies were described, comprising acquired antigen-presenting loss such as HLA class I or bêta-2-microglobulin deficiency [22, 23] or JAK deficiency leading to interferon-receptor pathway dysfunction [24, 25]. More investigations are needed to determine factors of resistance to second-course immunotherapy.

## 5. Conclusions

This retrospective study shows real-life data describing survival in patients with advanced melanoma after discontinuation of anti-PD-1 immunotherapy for other reason than progression. It shows a recurrence rate of 18.5%. There are reassuring data that suggest that discontinuation of anti-PD-1 immunotherapy may be proposed in the case of OR (including PR), even in case of SD. We suggest close follow-up during the first year after discontinuation of treatment. Usual poor prognostic factors do not appear to be correlated with the risk of recurrence; however, study power was low. There is a crucial need to understand the underlying factors which determine whether or not a patient will experience a long-lasting response to immunotherapy and, beyond this, to find robust criteria allowing physicians to best determine when to withdraw PD-1 inhibitors.

## Figures and Tables

**Table 1:** Patient characteristics at baseline.

Baseline characteristics	*n* = 65
*Age (year)*	64 (22–85)
<65 years	33 (50.8%)
>65 years	32 (49.2%)

*Sex*	
Men	42 (64.6%)
Women	23 (35.4%)

*ECOG*	
0	38 (58.5%)
1	22 (33.8%)
2	4 (6.1%)
3	1 (1.6%)

*Anti-PD-1 antibody*	
Pembrolizumab	49 (75.4%)
Nivolumab	16 (24.6%)

*BRAF status*	
Mutant	22 (33.8%)
Wild type	43 (66.2%)

*Albuminemia*	
Normal	53 (81.5%)
<35 g/l	2 (3.1%)
Unknown	10 (15.4%)

*LDH*	
< ULN	36 (55.4%)
> ULN	12 (18.4%)
Unknown	17 (26.2%)

*Prior systemic therapies*	
0	36 (55.4%)
1	20 (30.8%)
2	5 (7.7%)
3	3 (4.6%)
4	1 (1.5%)
Prior ipilimumab	14 (21.5%)
Prior BRAF ± MEK inhibitor	18 (27.7%)
Concurrent radiotherapy	12 (18.4%)
*Number of metastatic localizations*	
1	17 (26.2%)
2	22 (33.8%)
3	14 (21.5%)
>3	12 (18.5%)
Brain metastasis	11 (16.9%)

Values are *n* (%) or median (interquartile range). Abbreviations: ECOG PS, eastern cooperative oncology group performance status; PD-1, programmed cell death protein 1; LDH, lactate dehydrogenase; ULN, upper limit of normal.

**Table 2:** Clinical characteristics in patients who experienced a relapse.

Cause of discontinuation	Relapses (*n* = 12)		
	CR (*n* = 3)	SD/PD (*n* = 2)	AEs (*n* = 7)
*Age*			
<65 years	3	2	3
>65 years	0	0	4

*Sex*			
Men	2	1	2
Women	1	1	5

*BRAF status*			
Mutant	2	1	3
Wild type	1	1	4

*First-line treatment*			
Yes	1	1	3
No	2	1	4

*Prior ipilimumab*			
Yes	2	0	3
No	1	2	4

*Prior BRAF* *±* *MEK inhibitor*			
Yes	1	1	2
No	2	1	5

*Radiotherapy while on treatment*			
Yes	2	0	2
No	1	2	5

*TOT*			
<12 months	0	1	5
>12 months	3	1	2

*Relapse site*			
Known sites	1	1	4
New lesions	2	1	3

**Table 3:** Predictive factors associated with relapse in the anti-PD-1 cohort estimated with Cox regression model, University Hospital of Bordeaux, 2019.

Variable	Crude HR (95% CI)	*p* ^1^	Adjusted HR (95% CI)	*p* ^2^
Age	0.97 (0.94, 1)	0.065	0.97 (0.93, 1.01)	0.122
Women	2.28 (0.72, 7.2)	0.155	2.21 (0.64, 7.69)	0.211
TOT	0.97 (0.91, 1.04)	0.417	0.94 (0.85, 1.03)	0.178
Reason of discontinuation (ref. = toxicity)		0.445		
PR/SD	0.7 (0.14, 3.46)			
CR	0.43 (0.11, 1.67)			
BRAF_mutation	2.18 (0.7, 6.77)	0.184	1.95 (0.56, 6.80)	0.293
Number of metastasis	1.22 (0.83, 1.78)	0.331		
Cerebral metastasis	1.94 (0.58, 6.45)	0.302		
High LDH	3.6 (0.72, 17.89)	0.129		

^1^Crude *p* value, likelihood ratio test; ^2^adjusted *p* value, likelihood ratio test; TOT: time on treatment; CR: complete response; PR: partial response; SD: stable disease; LDH: lactate dehydrogenase.

**Table 4:** Comparison of subgroups according to cause of anti-PD-1 discontinuation in the anti-PD-1 cohort, University Hospital of Bordeaux, 2019.

	All patients, *n* = 65	CR group, *n* = 25	PR/SD group, *n* = 12	AEs group, *n* = 28	*p* value^1^
Median follow-up after introduction of treatment months (range)	36.5 (4.6–62.4)	40.5 (21–54.3)	40.4 (25.4–62.4)	29.7 (4.6–52.1)	0.015
Median TOT months (range)	14.1 (0.7–51.2)	16.8 (7.6–34.9)	21.2 (10.1–51.2)	7.2 (0.7–30.2)	<0.001
Median follow-up after discontinuation months (range)	15.7 (2.5–45.1)	22.3 (6.2–42.3)	11.3 (5.9–31.5)	12.7 (2.5–45.1)	0.144
Median time to best response months (range)	5.6 (0.7–32)	6.2 (2.5–21.4)	4.9 (2.8–27.8)	4.5 (0.7–32)	0.537
Relapses, *n* (%)	12 (18.5)	3 (12)	2 (16.7)	7 (25)	0.491
Median time before relapse months (range)	9 (1.9–40.9)	9.3 (4–11.8)	11.9 (10.9–12.8)	7.1 (1.9–40.9)	0.308
Second-course after relapse, *n* (%)	9 (13.8)	3 (12)	2 (16.7)	4 (14.3)	1

^1^ *p* value obtained with Kruskal–Wallis and Fisher tests for three groups equality. Abbreviation: TOT: time on treatment; CR, complete response; PR, partial response; SD, stable disease.

## Data Availability

The data used to support the findings of this study are available on request to the corresponding author.
